# Effects of Humidity and Surfaces on the Melt Crystallization of Ibuprofen

**DOI:** 10.3390/ijms130810296

**Published:** 2012-08-17

**Authors:** Dong-Joo Lee, Suyang Lee, Il Won Kim

**Affiliations:** Department of Chemical Engineering, Soongsil University, Seoul 156-743, Korea; E-Mails: dongjoo0428@naver.com (D.-J.L.); suyang.leee@gmail.com (S.L.)

**Keywords:** crystallization, ibuprofen, melt, humidity, surfaces

## Abstract

Melt crystallization of ibuprofen was studied to understand the effects of humidity and surfaces. The molecular self-assembly during the amorphous-to-crystal transformation was examined in terms of the nucleation and growth of the crystals. The crystallization was on Al, Au, and self-assembled monolayers with –CH_3_, –OH, and –COOH functional groups. Effects of the humidity were studied at room temperature (18–20 °C) with relative humidity 33%, 75%, and 100%. Effects of the surfaces were observed at −20 °C (relative humidity 36%) to enable close monitoring with slower crystal growth. The nucleation time of ibuprofen was faster at high humidity conditions probably due to the local formation of the unfavorable ibuprofen melt/water interface. The crystal morphologies of ibuprofen were governed by the nature of the surfaces, and they could be associated with the growth kinetics by the Avrami equation. The current study demonstrated the effective control of the melt crystallization of ibuprofen through the melt/atmosphere and melt/surface interfaces.

## 1. Introduction

Crystallization is a process where self-assembly is initiated at a molecular level during nucleation. The self-assembly of the molecules continues as crystals grow, obeying specific assembly rules defined by the various molecular interactions. The assembly rules are ultimately translated into the crystallographic structures. Often, molecules could form several crystallographic structures to show multiple metastable and stable phases, a phenomenon known as polymorphism [1]. Also, the assembly exhibited through the crystal growth can be directionally fine-tuned to express diverse crystal morphologies while maintaining the same crystallographic structures [2,3]. Since the molecular packing, that determines the polymorphs and morphology, has direct implications in the physical properties of the crystals [4], it is important in the usage of crystalline materials to discover various controlling parameters of the crystallization.

Controlled crystallization of active pharmaceutical ingredients could affect their bioavailability, processability, and stability. Bioavailability is usually a function of solubility, which can be modulated by the molecular packing [5,6]. Processability of crystal particles is represented by their flowability and packing properties, which are decidedly affected by the crystal morphology [7,8]. The stability issue arises most often in metastable polymorphs, nanoparticles, and kinetically stabilized amorphous phases [1,9–11].

In the present study, melt crystallization of ibuprofen (IBU) was investigated. Melt crystallization is essentially an amorphous-to-crystal transformation governed by supercooling, defined as the difference between the melting and crystallization temperatures. During typical melt crystallization, two existing interfaces are melt/surface and melt/atmosphere. Herein, we studied the effects of various surfaces and humidity to alter these interfaces and ultimately control the molecular assembly process of IBU.

## 2. Results and Discussion

### 2.1. Effects of Humidity on the Melt Crystallization of IBU

**Figure 1:**
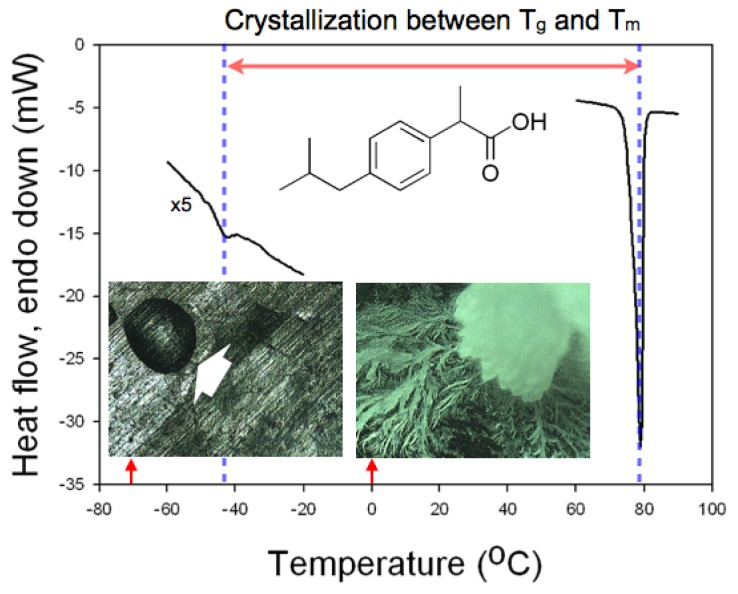
A differential scanning calorimetry (DSC) thermogram of ibuprofen (IBU) showing *T*_g_ at −42.3 °C and *T*_m_ at 78.9 °C. The two optical micrographs were taken when IBU melt was placed on Al at −70 °C (left) and 0 °C (right) for 12 h.

Differential scanning calorimetry (DSC) was performed to confirm the temperature range that could be utilized for the crystallization of IBU. The two limiting points that defined the temperature range were taken as the glass transition temperature and the melting point of IBU [12]. The former decided the temperature below which the mobility of the IBU molecules would be prohibitively low to initiate the molecular self-assembly that would be necessary to form IBU nuclei. The latter defined the temperature where the supercooling, the driving force for the melt crystallization, would be nil. The glass transition temperature and the melting point of IBU were found as −42.3 °C and 78.9 °C, respectively, with the scanning rate of 10 °C/min (Figure 1), which was in accordance with the previous studies [13,14]. For example, as shown in the micrographs in Figure 1, when IBU melt was placed at −70 °C, no crystallization occurred even after 12 h (amorphous IBU denoted by a white arrow), while crystallization of IBU melt was complete at 0 °C under the same conditions otherwise. Based on the DSC study, we chose room temperature (18–22 °C) for the crystallization experiments with controlled relative humidity (RH) because it was about the center of the glass transition temperature and the melting point, where sufficient mobility and supercooling were provided.

**Figure 2:**
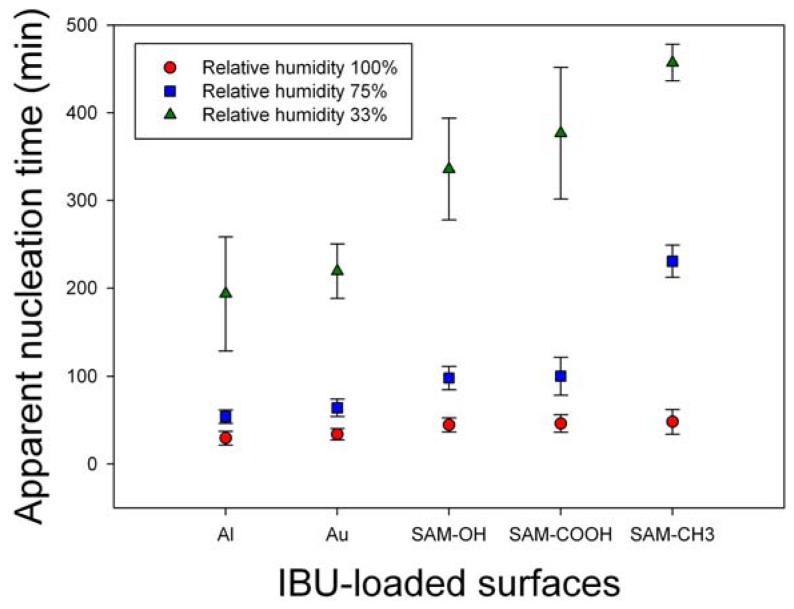
Nucleation time of IBU crystals at room temperature and relative humidity 33%, 75%, and 100% on various surfaces.

The effects of humidity on IBU melt crystallization were monitored in terms of nucleation, crystallinity, crystal phase, and morphology. First, the effect on the nucleation was summarized in Figure 2. Nucleation time was monitored at RH 33%, 75%, and 100%, and the crystallization was on Al, Au, and self-assembled monolayers with –OH, –COOH, and –CH_3_ functional groups (SAM–OH, SAM–COOH, and SAM–CH_3_, respectively). Each result was the average of eight independent experiments. On all surfaces, the observation time of the initial nucleation was inversely proportional to RH, *i.e.*, the nucleation time was in the order, RH 100% < 75% < 33%. Also, the nucleation time was similar for all surfaces at RH 100%, and it was relatively shorter on metal surfaces and longer on SAM surfaces (especially SAM–CH_3_) at RH 75% and 33%. Overall, we could conclude that humidity, probably through the adsorbed water molecules at the melt/atmosphere interface, acted on the IBU melts to initiate the molecular assembly and start the nucleation process. Although the exact mechanism through which the water molecules affected the metastable IBU melts is not clear at this point, it could be through the increased instability. The adsorbed water molecules on the metastable IBU melts would replace some of the existing IBU melt/air interface with the melt/water interface. The new interface is most likely less favorable since water is immiscible with IBU melt, and it is a very poor solvent of IBU (solubility 0.011 mg/mL at 25 °C and pH 7.4) [15,16]. Therefore, the newly formed and unfavorable interface would prompt the metastable melts to nucleate a denser crystal phase to reduce the interfacial area of IBU/water. The nucleating effect of water was also confirmed by observing the immediate crystallization when the IBU melt was in contact with liquid water (data now shown).

**Figure 3:**
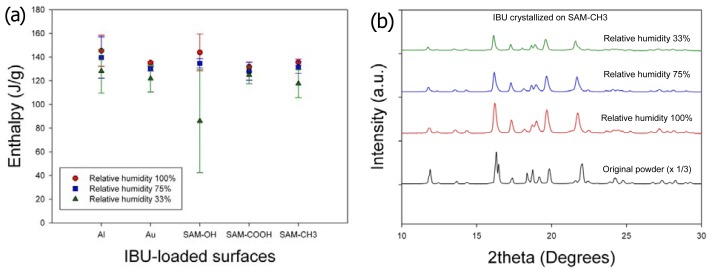
(**a**) The heat of fusion of the IBU crystals formed on various surfaces at room temperature and relative humidity 33%, 75%, and 100%; (**b**) powder X-ray diffraction (PXRD) patterns of IBU crystals formed on SAM-CH_3_ at room temperature and varied relative humidity, compared with commercial IBU powders.

**Figure 4:**
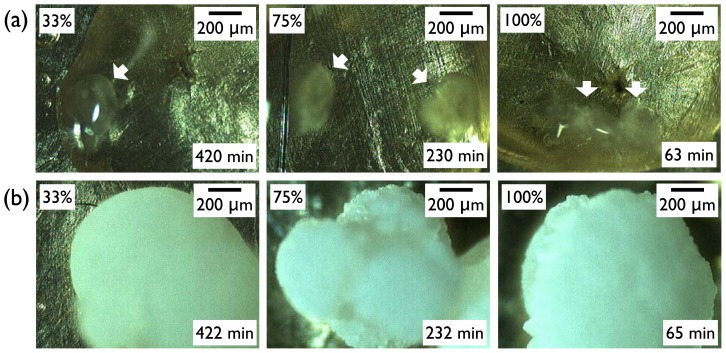
*In situ* optical micrographs showing IBU crystal formation on SAM–CH_3_ at room temperature and relative humidity 33%, 75%, and 100%: (**a**) initial appearance of crystals (white arrows); (**b**) near complete crystal formation after 2 more min.

Second, the effects of humidity on the crystallinity and crystal phase (or polymorph) of IBU were minimal (Figure 3). The melting enthalpy of the samples, 1 day after crystallization, was summarized in Figure 3a (three independent experiments). Slight increase in the heat of fusion along with humidity increase was within the range of standard deviations, and any correlation with the surfaces was hard to establish. (Note that the melting enthalpy of the original powder was 141.0 ± 5.9 J/g.) The melting points of all the obtained crystals were located in the range *ca.* 75 °C–78 °C, suggesting that the obtained crystals were of the same crystal polymorph. (Note that the melting point of the original powder was 78.9 °C.) This was also confirmed by powder X-ray diffraction (PXRD) patterns, as the characteristic diffraction peaks were identical among the purchased powders and all the recrystallized samples on all surfaces. Representative results were shown in Figure 3b for the IBU recrystallized on SAM–CH_3_ along with the originally purchased IBU powders, and the results were the same for the IBU recrystallized under all the other conditions. Finally, the effect of humidity on the IBU crystal morphology was negligible. Again, the representative case was shown for the IBU recrystallized on SAM–CH_3_ (Figure 4). Top pictures (Figure 4a) were taken as soon as the turbid regions appeared within the clear area of IBU melts. Note that the *in situ* OM observation was made through a clear polystyrene chamber (diameter 9 cm, height 1.5 cm, SPL Lifesciences) connected through a rubber vacuum tube to a glass desiccator (diameter 14 cm, height 16 cm) of the controlled RH. Within a few minutes of initial crystal formation, the crystal growth appeared to complete as shown in the bottom pictures (Figure 4b). The morphology was more or less spherical for the IBU recrystallized on SAM–CH_3_ irrespective of the relative humidity. (Note that the morphologies of the IBU crystals were not the same among the various crystallization surfaces, and the results were summarized in the next section. In the current section, the effect of the various surfaces was intentionally omitted to focus on the influence of humidity.) From these results, we could conclude that the effects of humidity was largely limited to the nucleation of IBU crystals, and the crystallinity, crystal phase, and morphology of IBU were on the whole invariant with the change of relative humidity.

### 2.2. Effects of Surfaces on the Melt Crystallization of IBU

To study the effects of the various surfaces, the melt crystallization of IBU was performed at −20 °C, rather than room temperature as was in the pervious section. This condition was chosen to make it easy to monitor the growth behavior on the various surfaces because it ensured a relatively slower crystal growth after nucleation, probably due to the combined effects of the low temperature and reduced humidity. The very low atmospheric water content also made it possible to examine the effects of surfaces with a minimal influence of humidity. (The water content is only 0.23 g/kg dry air at this condition (−20 °C, RH 36%) compared to 4.75 g/kg dry air at 20 °C and RH 33% [17]). Also, the IBU crystal morphology was found to be virtually the same for the crystallization at −20 °C, 0 °C, and room temperature for the same surface.

In this experiment, multiple IBU melts on the same type of surfaces were placed in the −20 °C freezer in the beginning, and each sample was taken out at a given crystallization time to microscopically observe the crystalline area out of the entire melt area. Then, the crystallization behavior was analyzed through the Avrami equation:

$$X_{\text{c}}(t)=1-\text{exp}(-{Kt}^{n})\;\text{or ln}[-\text{ln}(1-X_{\text{c}}(t))=n\text{ln}t+\text{ln}K]$$

where *X*_c_(*t*) is the relative crystallinity at time *t*, *n* is the Avrami exponent, and *K* is the rate constant. Since the Avrami equation is based on the spatial profile of molecular assembly on the crystal surfaces, the Avrami-type analysis of the crystallization kinetics provides important information on the assembly and the resulting crystal morphology [12,18].

**Figure 5:**
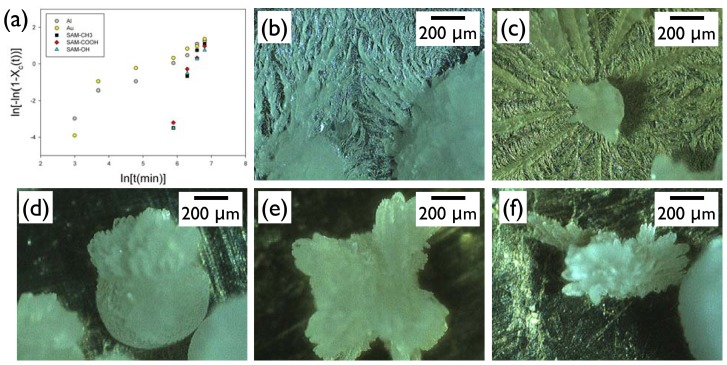
(**a**) An Avrami plot of IBU crystallization at −20 °C on various surfaces. Corresponding optical micrographs at the end of the crystallization was shown for (**b**) Al; (**c**) Au; (**d**) SAM-CH_3_; (**e**) SAM-COOH; and (**f**) SAM-OH.

While the microscopic measurement of the crystallinity in the middle of crystal growth was difficult to assess accurately because of the three-dimensional nature of the crystals, projected area of the crystals out of the entire melt region was roughly taken as the relative crystallinity, *X*_c_. This analysis clearly revealed that the Al and Au surfaces exhibited completely different behavior from the SAM surfaces (Figure 5a). On these high-energy metal surfaces, the crystallization started more quickly (<20 min) than on the SAM surfaces, and it appeared to have two sequential stages: (i) initially rapid growth; (ii) later slower propagation stages. The Avrami exponent for the IBU growth on Al and Au of the initial stage was *ca.* 2.2–4.3, and that of the later stage was 0.76–1.4. In contrast, the crystallization started only after about 360 min on the SAM surfaces, and it was complete relatively quickly. The Avrami exponents for the IBU growth on the SAM-OH, SAM-COOH, and SAM-CH_3_ were *ca.* 4.5–5.2. Note that the theoretical Avrami exponents for the spherical growth are 3 or 4 depending on the preexistence or constant formation of the nuclei, for the fibrillar growth less than 1 or 2, and for sheaf-like growth more than 5 [12]. The analysis of the growth kinetics based on the Avrami equation was in good agreement with the morphology observation. The IBU on Al or Au (Figure 5b,c) consisted of the approximately spherical crystals surrounded by the fibrillar structures closely attached to the metal surfaces. The IBU on SAM surfaces (Figure 5d–f) only exhibited bulk crystals, of which end structures were often bundled needles or sheaf-like, and no fibrillar formation was found on the SAM surfaces. From these results, we could conclude that the surfaces affected both nucleation and growth of IBU crystals. The nucleation time was relatively faster on the metal surfaces, which was in accordance with the results of Section 2.1 (e.g., see the low-humidity case). The growth kinetics was profoundly different between metals and SAMs. The former surfaces induced initially spherical and later fibrillar growth. The latter formed a combined morphology of spherical and sheaf-like crystals. This difference seemed to arise from the balanced interactions of the IBU melt with the surfaces and the growth front of the crystals [19]. Experiments and calculations are currently underway to explore the nature of these interactions.

## 3. Experimental Section

IBU was purchased from Sigma (>98%). Its thermal behavior was studied using differential scanning calorimetry (DSC: DSC821^e^, Mettler-Toledo; calibrated for temperature and enthalpy using indium). Also, its recrystallization behavior was studied on Al, Au, and three different self-assembled monolayers (SAMs). Al crucibles (ME-27331, Mettler-Toledo) were utilized for all surfaces to make their roughness uniform. Al surface was used after successively cleaning with acetone (>99.8%, Daejung Chemical, Seoul, Korea), ethanol (>99.9%, Samchun Chemical, Seoul, Korea), and deionized water (resistivity > 18.2 MΩ-cm; Direct-Q, Millipore). Au surface was prepared on the cleaned Al by sputter-coating Au (99.99%–99.999%, Taewon Scientific Co., Seoul, Korea) using a Cressington Sputter Coater 108 (*ca.* 6 μm, 20 min coating time at 10 mA and 35 mbar) [20]. SAM surfaces with –CH_3_, –OH, and –COOH functional groups (SAM–CH_3_, SAM–OH, and SAM–COOH) were prepared by treating the Au surfaces in 1.0 mM (ethanol) solutions of 1-decanethiol (96%, Aldrich), 11-mercapto-1-undecanol (97%, Aldrich), and 11-mercaptoundecanoic acid (95%, Aldrich), respectively [20]. After 24 h, the SAM surfaces were cleaned with ethanol and dried with N_2_ (>99.9%, Seoul Special Gas, Seoul, Korea) before further usage.

Crystallization of IBU was performed by first preparing IBU melt and then recrystallizing the melt at appropriate conditions. The IBU melt was prepared on the various surfaces by melting the purchased IBU powders (2–3 mg) using a hot stage (FP82HT, Mettler-Toledo). Temperature of the hot stage was increased from 30 °C to 100 °C (10 °C/min), and it was cooled to 30 °C (*ca.* 14 min using an internal fan of the hot stage). The IBU samples were then subject to the environments of controlled temperature and humidity for further observation of recrystallization. Recrystallization temperature was −70 °C, −20 °C, 0 °C, or room temperature (*ca.* 18–22 °C). The −70 °C freezer was a DF8514 from Ilshin Biobase (Seoul, Korea), and the −20 °C and 0 °C conditions were obtained using a R-A104GDA from LG Electronics (Seoul, Korea). The relative humidity for the room temperature experiments was controlled as 33%, 75%, and 100% by using saturated MgCl_2_(aq) (magnesium chloride hexahydrate, 99.0%–102.0%, Sigma-Aldrich), saturated NaCl(aq) (sodium chloride, >99.5%, Sigma-Aldrich), and deionized water [21].

Morphology of the recrystallized IBU was observed with optical microscopy (OM: BX51, Olympus) in the reflectance mode under cross polarization. The crystalline nature of the recrystallized IBU was also analyzed by powder X-ray diffraction (PXRD) and DSC. PXRD was performed using a D2 Phaser diffractometer (Bruker AXS). CuKα radiation (*λ* = 1.5406 Å) at 30 kV and 10 mA was used to scan the 2*θ* region of 5°–40° with a scanning rate of 1°/min. DSC was done again using a DSC821^e^ (Mettler-Toledo) with a scanning rate of 10 °C/min.

## 4. Conclusions

In summary, the effects of humidity and surfaces were studied on the melt crystallization of ibuprofen to enable the controlled crystallization through melt/atmosphere and melt/surface interfaces. The humidity was effective in controlling the nucleation time. High humidity influenced the initiation of crystal formation probably through the local development of unfavorable melt/water interfaces. The humidity could control the IBU nucleation independently with minimal effects on the crystallinity, polymorphs, and crystal morphology. The examined surfaces, which could be broadly categorized as high-energy metal and functionalized SAM surfaces, affected mostly the morphology of the IBU crystals, and the crystal morphology was in agreement with the Avrami analysis of the crystal growth. Our results demonstrated that the melt/atmosphere and melt/surface interfaces could be effectively utilized to control the melt crystallization without any additional additives.

## References

[1] Bernstein J Polymorphism in Molecular Crystals.

[2] Buckley H.E. (1951). Crystal Growth.

[3] Kim I.W., Collino S., Evans J.S. (2012). Cooperative modulation of mineral growth by prismatic-associated asprich sequences and Mg(II). Int. J. Mol. Sci.

[4] Mullin J.W. (2001). Crystallization.

[5] Blagden N., de Matas M., Gavan P.T., York P. (2007). Crystal engineering of active pharmaceutical ingredients to improve solubility and dissolution rates. Adv. Drug Deliv. Rev.

[6] Rodríguez-Spong B., Price C.P., Jayasankar A., Matzger A.J., Rodríguez-Hornedo N. (2004). General principles of pharmaceutical solid polymorphism: A supramolecular perspective. Adv. Drug Deliv. Rev.

[7] Chow K., Tong H.H.Y., Lum S., Chow A.H.L. (2008). Engineering of pharmaceutical materials: An industrial perspective. J. Pharm. Sci.

[8] Lee M.K., Lee H., Kim I.W., Lee J. (2011). Novel polymorphic form of adefovir dipivoxil derived from polymer-directed crystallization. Pharmazie.

[9] Gu C.-H., Grant D.J.W. (2001). Estimating the relative stability of polymorphs and hydrates from heats of solution and solubility data. J. Pharm. Sci.

[10] Shegokar R., Müller R. (2010). Nanocrystals: Industrially feasible multifunctional formulation technology for poorly soluble actives. Int. J. Pharm.

[11] Yu L. (2001). Amorphous pharmaceutical solids: Preparation, characterization and stabilization. Adv. Drug Deliv. Rev.

[12] Gedde U.W. (1995). Polymer Physics.

[13] Manish M., Harshal J., Anant P. (2005). Melt sonocrystallization of ibuprofen: Effect on crystal properties. Eur. J. Pharm. Sci.

[14] Dudognon E., Danède F., Descamps M., Correia N.T. (2008). Evidence for a new crystalline phase of racemic ibuprofen. Pharmaceut. Res.

[15] Garzón L.C., Martínez F. (2004). Temperature dependence of solubility for ibuprofen in some organic and aqueous solvents. J. Solut. Chem.

[16] Paradkar A.R., Maheshwari M., Ketkar A.R., Chauhan B. (2003). Preparation and evaluation of ibuprofen beads by melt solidification technique. Int. J. Pharm.

[17] Climate calculator.

[18] Kim Y.C. (2006). Effect of maleated polyethylene on the crystallization behavior of LLDPE/clay nanocomposites. Polym. J.

[19] Chung J., Kim B.J., Chung H., Kim I.W. (2011). Melt crystallization of ibuprofen on surfaces patterned by a Nd: YAG laser. Korean J. Chem. Eng.

[20] Chung J., Kim I.W. (2011). Oriented crystallization of xanthine derivatives sublimated on self-assembled monolayers. Korean J. Chem. Eng.

[21] Sjöqvist M., Boldizar A., Rigdahl M. (2010). Processing and water absorption behavior of foamed potato starch. J. Cell. Plast.

